# Nutritional and Nonnutritional Content of Underexploited Edible Seaweeds

**DOI:** 10.1155/2022/8422414

**Published:** 2022-10-15

**Authors:** Rabia Alghazeer, Hesham El Fatah, Salah Azwai, Sana Elghmasi, Maammar Sidati, Ali El Fituri, Ezdehar Althaluti, Ftaim Gammoudi, Ervia Yudiati, Nadia Talouz, Ghalia Shamlan, Ammar AL-Farga, Wafa S. Alansari, Areej A. Eskandrani

**Affiliations:** ^1^Chemistry Department, Faculty of Sciences, University of Tripoli, Tripoli, Libya; ^2^Botany Department Faculty of Science, Ain Shams University, Cairo, Egypt; ^3^Department of Microbiology and Parasitology, Faculty of Veterinary Medicine, University of Tripoli, Tripoli, Libya; ^4^Department of Biochemistry, Faculty of Medicine, University of Tripoli, Tripoli, Libya; ^5^Marine Biotechnology Department, Marine Biology Research Center, Tajura-East of Tripoli, Tripoli, Libya; ^6^Department of Marine Chemistry and Physics, Marin Biology Research Center, Tajura-East of Tripoli, Tripoli, Libya; ^7^Department of Marine Science, Faculty of Fisheries and Marine Science, Universitas Diponegoro, Jl., Indonesia; ^8^Department of Botany, Faculty of Science, University of Tripoli, Tripoli, Libya; ^9^Department of Food Science and Nutrition, College of Food and Agriculture Sciences, King Saud University, Riyadh 11362, Saudi Arabia; ^10^Biochemistry Department, Faculty of Science, University of Jeddah, Jeddah 21577, Saudi Arabia; ^11^Chemistry Department, Faculty of Science, Taibah University, Medina 30002, Saudi Arabia

## Abstract

Macroalgae are a valuable source of highly bioactive primary and secondary metabolites that may have useful bioapplications. To investigate the nutritional and nonnutritional contents of underexploited edible seaweeds, proximate composition, including protein, fat, ash, vitamins A, C, and E, and niacin, as well as important phytochemicals, including polyphenols, tannins, flavonoids, alkaloids, sterols, saponins, and coumarins, were screened from algal species using spectrophotometric methods. Ash content ranged from 3.15–25.23% for green seaweeds, 5–29.78% for brown algae, and 7–31.15% for red algae. Crude protein content ranged between 5 and 9.8% in Chlorophyta, 5 and 7.4% in Rhodophyta, and between 4.6 and 6.2% in Phaeophyceae. Crude carbohydrate contents ranged from 20 to 42% for the collected seaweeds, where green algae had the highest content (22.5–42%), followed by brown algae (21–29.5%) and red algae (20–29%). Lipid content was found to be low in all the studied taxa at approximately 1–6%, except for *Caulerpa prolifera* (Chlorophyta), which had a noticeable higher lipid content at 12.41%. These results indicated that Phaeophyceae were enriched with a high phytochemical content, followed by that of Chlorophyta and Rhodophyta. The studied algal species contained a high amount of carbohydrate and protein, indicating that they could be considered as a healthy food source.

## 1. Introduction

Marine algae are a valuable source of highly bioactive primary and secondary metabolites that may have potential bioapplications in the development of new industrial, pharmaceutical, and food applications. Several active compounds from natural sources have shown reduced side effects and are of great interest because of their very low cytotoxicity [1].

The nutritional value of algae is very important as it has been used as a part of the diet in many countries, particularly those in Asia [2, 3]. The variety of chemical components in algae and their quantity depends on many factors such as species, maturity, and environmental conditions [4]. Algae are nutritionally important with a high level of vital nutrients, including polysaccharides, polyunsaturated fatty acids, proteins, and amino acids, as well as dietary fiber, vitamins, and minerals [5–7]. In addition, algae contain a wide variety of nutritional minerals, including iodine, potassium, calcium, magnesium, phosphorus, iron, and zinc [8]. One of the most valuable nutritional properties of algae is related to their high content of polysaccharide.

In addition to their nutritional value, seaweeds contain various nonnutritional compounds that recently have been the subject of considerable scientific and therapeutic interest [9]. The major bioactive compounds of marine algae include phenolics, phlorotannins, terpenes, terpenoids, alkaloids, tannins, and flavonoids [10, 11]. Algae also contain antioxidants, including polyphenols, carotenoids, and flavonoids [12, 13], while compounds, such as rutin, quercetin, and kaempferol, as well as flavonoids, have been identified in many algal species [14]. In addition, several marine algae have been assessed in *in vitro* and *in vivo* investigations for their anticancer activity [15, 16].

A Libyan study has reported the phytochemical analysis and antioxidant and antimicrobial effect of several seaweeds [17]; however, the availability of pharmaceutical data from seaweeds is still rare in comparison with that from plants. In this study, we undertook a qualitative and quantitative analysis of many of the nonnutritive and nutritive compounds in 20 different algal species collected from different areas of Libya.

## 2. Materials and Methods

### 2.1. Sample Collections

**Figure 1:**
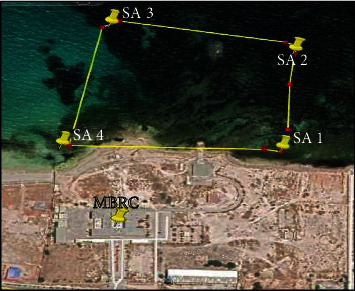
Algae collection site locations. (MBRS: Marine Biology Research Center).

Twenty-four species were studied from three groups of algae. These included Chlorophyta (green algae): *Caulerpa prolifera* (collected in 2021 from Farwa Island, Zuwara, 90 km west of Tripoli) and *Codium tomentosum*, *Ulva compressa* (formerly *Enteromorpha compressa*), *Ulva intestinalis* (formerly *Enteromorpha intestinalis*), *Ulva linza* (*Enteromorpha linza*), *Flabellia petiolata*, *Halimeda tuna*, and *Ulva lactuca*; Phaeophyceae (brown algae): *Cladostephus spongiosus*, *Cystoseira compressa*, *Ericaria amentacea* (formerly *Cystoseira stricta*), *Dictyota dichotoma*, *Halopteris scoparia*, *Padina pavonica*, *Petalonia fascia*, and *Sargassum hornschuchii*; and Rhodophyta (red algae): *Asparagopsis taxiformis*, *Ceramium virgatum* (formerly *Ceramium rubrum*), *Corallina officinalis*, *Pterocladiella capillacea* (formerly *Gelidium capillaceum*), *Gracilariopsis longissima* (formerly *Gracilaria verrucosa*), *Hypnea musciformis*, *Jania rubens*, and *Osmundea pinnatifida* (formerly *Laurencia pinnatifida*) were collected on 2021 from the western coast of Libya (SA 01, N 32°53'45.47 E 13°21'3.16; SA 02, N 32°53'51.95 E 13°21'4.25; SA 03, N 32°53'54.19 E 13°20'54.10; SA 04, N 32°53'46.23 E 13°20'50.90) (Figure 1). The algal samples were taxonomically identified at the Marine Biology Research Center, Tajura, East of Tripoli, Libya.

The collected algae were cleaned with sea water to remove all the extraneous matter (epiphytes and necrotic parts) and brought to the laboratory in plastic bags. Thereafter, the algae were thoroughly washed with tap water, followed with distilled water before being dried at room temperature in the shade for 7–14 days. The dried samples were grounded thoroughly into fine powder using a kitchen blender. The powdered samples were then stored at 4°C.

### 2.2. Phytochemical Screening

The tested extracts were screened for sterols, alkaloids, phenolic compounds, tannins, saponins, flavonoids, glycosides, coumarins, and quinones. Phytochemical screening of the extracts was performed according to the standard method described by Harborne [18].

### 2.3. Proximate Analysis

Carbohydrate, protein, fat, ash, and moisture content were estimated according to the procedure of the Association of Official Analytical Chemists [19].

### 2.4. Determination of Vitamin Contents

Vitamin A, C, and E and niacin levels in the extracts were determined according to the methods described by Okwu and Ndu [20].

### 2.5. Quantitative Determination of Phytochemicals

Total phenolic content was estimated according to the Folin–Ciocalteu colorimetric method by Singleton et al. [21] using gallic acid as the standard. Total flavonoid content was estimated as described by Zhishen et al. [22] using rutin as the standard. Total tannin content was determined as detailed by Julkunen-Tiitto [23] using tannic acid as the standard. Total alkaloid content was determined as described by Shamsa et al. [24] and Sharief et al. [25] using atropine as the standard. Total coumarin content was estimated following the standard methods by Buragohain [26] and de Osório and Martins [27] using coumarin as the standard. Total steroid content was estimated according to Devanaboyina et al. [28] using cortisone as the standard.

## 3. Results and Discussion

### 3.1. Phytochemical Analysis

Important phytochemicals, such as polyphenols, tannins, flavonoids, alkaloids, sterols, saponins, and coumarins, were screened from algal species collected from the western coast of Libya. The phytochemical contents obtained from the extraction of the collected algae are shown in Table 1. This analysis showed that Phaeophyceae were highly enriched in phytochemicals, followed by Chlorophyta and Rhodophyta (Table 1).

### 3.2. Proximate Primary Composition

**Figure 2:**
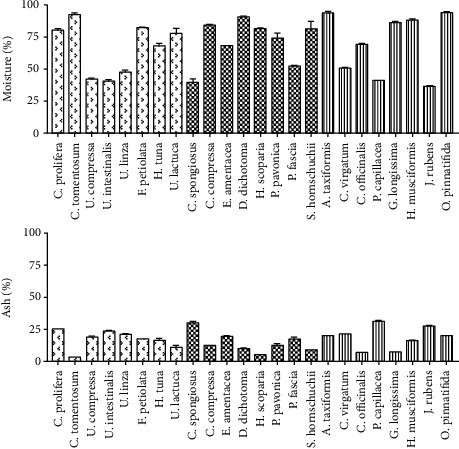
Proximate composition of moisture and ash expressed as percentage (%) of seaweeds collected from the Tripoli coastline.

**Figure 3:**
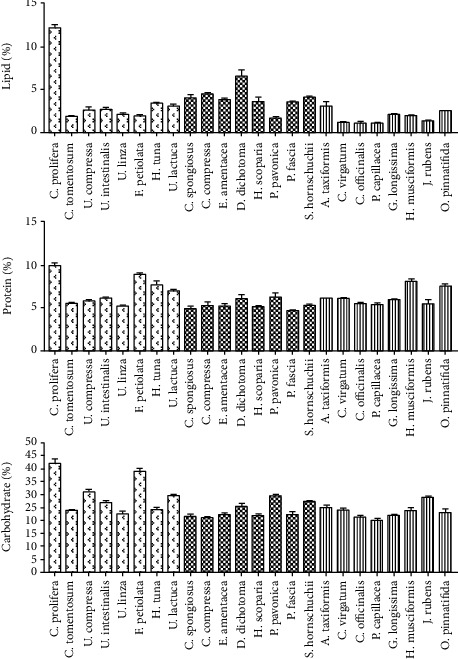
Proximate composition of proteins, fats, and carbohydrates expressed as percentage (%) of seaweeds collected from the Tripoli coastline.

The proximate composition of the dried seaweeds collected from Tripoli coastline is shown in Figures 2 and 3, with the moisture and ash shown in Figure 2. In Chlorophyta, the moisture content of the collected macroalgae was between 40.50 and 92.61%. *Ulva* spp. had the lowest levels of moisture content of approximately 40–47% after drying, while *C. tomentosum* had the highest value at 92.6%. The brown seaweed *C. spongiosus* had the lowest moisture level (39.77%) after drying, while *D. dichotoma* had the highest (90.55%) in Phaeophyceae. In the Rhodophyta, *J. rubens* had the lowest moisture content (36.56%), while *A. taxiformis* and *O. pinnatifida* had the highest content (93.57 and 93.82%, respectively). The ash content ranged from 3.15 to 25.23% for green seaweeds, with *C. tomentosum* and *C. prolifera* having the lowest and the highest values, respectively. For brown algae, *H. scoparia* had the lowest ash content (approximately 5%), and *C. spongiosus* had the highest (29.78%). For red algae, *P. capillacea* had the highest ash content (31.15%), while *C. officinalis* and *G. longissima* had the lowest (7%). We found that moisture contents were relatively high for most of the collected seaweeds. Wan et al. [29] observed similar results and determined that the moisture content from green, red, and brown species ranged from 64.9 to 94%. Lower residual moisture contents have been reported by other researchers using other methods such as oven-drying at 60°C or freeze-drying [30, 31]. The higher moisture content recorded in this study could be attributed to the drying method used for the algae (air-drying). Higher drying temperatures may reduce drying time and cost, but several compounds (e.g., vitamins, proteins, unsaturated fatty acids, phenols, and carotenoids) would be vulnerable to degradation during the drying process [32, 33]. The optimal method for drying the seaweeds should be used to obtain a high proximate composition, as the removal of water from seaweeds is a necessary step in maintaining their quality as a food or in their proximate composition [34]. The high ash content obtained in the collected seaweeds may be due to the collection of the algal samples during low-temperature seasons [35]. Furthermore, a high level of ash content is associated with the amount of mineral elements [34, 35].

The crude carbohydrate contents ranged from 20 to 42% of the collected seaweeds where green algae had the highest content with 22.5–42%, followed by brown algae and red algae with approximately 21–29.5% and 20–29%, respectively (Figure 3). *C. prolifera* and *U. linza* showed the lowest and the highest values in Chlorophyta, respectively. There was little variation in the carbohydrate contents between the Phaeophyta and Rhodophyta, with *C. compressa* and *P. capillacea* having the lowest value of around 20% and *P. pavonica* and *J. rubens* having the highest contents at 29% in brown and red algae, respectively. High carbohydrate content was observed from macroalgal species in several studies [36, 37]. These relatively high carbohydrate contents in green algae suggest that they could be an important source of phycocolloids in food and industrial uses. These results were similarly observed in other studies [31, 38].

The crude protein content differed widely across groups of algae with low concentrations between 5 and 9.8% in Chlorophyta, 5–7.4% in Rhodophyta, and 4.6%–6.2% in Phaeophyceae (Figure 3). Wells et al. [39] recorded that among the marine macroalgae, the red and green algae often contain high levels of protein (as % dry weight) in contrast to lower levels in most brown algae. The protein content was moderately low compared with those in other macroalgae and agrees with the results from other studies [31, 34, 38]. In contrast, Wan et al. [29] recorded the highest protein content in seaweeds from the Rhodophyta division, including *C. crispus*, *Gracilariopsis*, and *Pyropia* species. Small variations in the crude protein content of studied macroalgae could be because of similar environmental conditions and geographical collection sites [40, 41]. In addition, during seasons of nutrient limitation (for instance, the summer season in coastal waters) the protein content of macroalgal decreases, and the relative proportions of amino acids change [39, 42].

Macroalgal species have a relatively low lipid content with values of <5% w/dry weight [43]. Lipids in marine macrophytes are usually phospholipids and glycolipids [44]. Low lipid contents were observed in all the studied taxa at approximately 1%–6%, except for *C. prolifera*, which had the highest lipid content at 12.405% (Figure 3). In agreement with the observed results, Pirian et al. [35] stated that the higher lipid contents were associated with the green algae *Caulerpa sertularioides*, *C. racemosa*, and *Bryopsis corticulans* found in the Persian Gulf. For the brown algae, *D. dichotoma* had the highest lipid content at 6.50% (Figure 3). These results were similar to those recorded by McDermid and Stuercke [45] who found that *Dictyota acutiloba* and *Dictyota sandvicensis* had a total lipid content (16.1 ± 0.1 and 20.2 ± 0.1% dry weight). However, Miyashita et al. [46] stated that brown algal species found in temperate seas produced more lipids than those growing in tropical seas. Biancarosa et al. [47] also observed that brown species have a higher lipid content compared with those of green species.

### 3.3. Secondary Metabolite Composition

Algal seaweeds are rich in vitamins [39, 48]. Algae are a source of water-soluble vitamin B2 (riboflavin), B12 (cobalamin), and C (ascorbic acid) and lipid-soluble vitamin E (*α*-, *β*-, *γ*-, and *δ*-tocopherol, and *α*-, *β*-, *γ*-, and *δ*-tocotrienol) [29].

**Figure 4:**
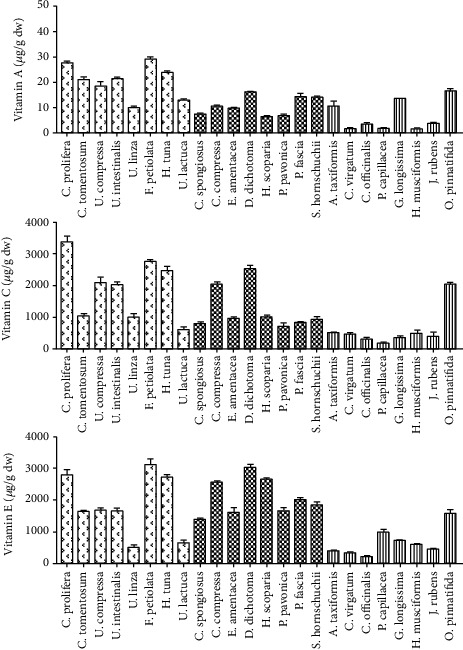
Proximate composition of vitamins, expressed as *μ*g/g dw of seaweeds collected from the Tripoli coastline. dw: dry weight.

The results of this study showed that chlorophyte and Phaeophyceae are rich in vitamin A and C. The green algae *F. petiolata* had the highest vitamin A content in all studied taxa, whereas the red algae *C. rubrum* and *H. musciformis* had the lowest content (Figure 4). Higher values of vitamin A in green algae may be due to their rich *β*-carotene content (provitamin A) as compared with that in other algal groups [49].

Vitamin E from seaweeds can be especially important in aquaculture feeds as this can serve as an internal antioxidant [29]. We found that brown and green algae had a higher content of vitamin E as compared with that in red algae (Figure 4). *F. petiolata* and *D. dichotoma* had the highest content of vitamin E from chlorophyte and Phaeophyta, respectively, while the red seaweed *Corallina officinalis* had the lowest content of vitamin E. These results agreed with earlier reports that stated that brown algae contained higher levels of vitamin E content followed by green and red algae [50].

### 3.4. Phenolics

Polyphenols have been widely described in plants and algae, and phenolic compounds have gained a significant attention because of their biological effects: antioxidant, antiproliferative, antimicrobial, antiallergic, antidiabetic, and neuroprotective actions [51–53], while others are known for either or both their toxicological effects and antinutritional properties [29]. The phenolic compounds found in macroalgae vary from simple molecules, such as phenolic acids or flavonoids, to the more complex phlorotannin polymeric structures.

Algae phenolic concentration is dependent on several factors, such as species, seasonal variations, and environmental conditions [54]. Phenolic compounds are considered as one of the most effective antioxidants in marine algae [55, 56]. We found that phenols were relatively low in chlorophytes and rhodophytes, where *U. lactuca* and *J. rubens* had the lowest values (0.66 ± 0.03 and 0.54 ± 0.03 mg GAE/gdw) (Table 2), whereas *C. prolifera* and *O. pinnatifida* had the highest values (3.46 ± 0.22 and 3.35 ± 0.17 mg GAE/gdw) from green and red algae, respectively. Brown algae had a relatively higher content of phenols compared with those in green algae, ranging between 0.65 ± 0.05 mg GAE/gdw in *Ericaria amentacea* and 3.31 ± 0.10 mg GAE/gdw in *D. dichotoma*. The higher total phenolic content resulted in higher antioxidant capacity. These results agreed with Chia et al. [57] who recorded those brown seaweeds to have a higher content of phenolic compounds compared with that in green seaweeds and that this may be due to the presence of phlorotannins, bipolar polyphenols that are commonly found in brown seaweeds.

Flavonoids are one of the most diverse and widespread groups of natural products and are probably the most important natural phenolics. The flavonoid content in red seaweeds was low and ranged from 0.49 to 14.84 mg RE/gdw. In green seaweeds, the flavonoid content varies from 4.78 mg RE/gdw in *U. lactuca* to 29.11 mg RE/gdw in *F. petiolata*. The highest flavonoid content was found in brown algae, which ranged between 6.86 and 32.38 mg RE/gdw, where *D. dichotoma* had the highest value at 32.38 mg RE/gdw (Table 2). Although the samples were collected during the same season, there was significant difference in their flavonoid content. This change in flavonoid content may be due to the variation in physicochemical parameters, such as salinity among the collected stations or environmental conditions [58].

In regard to the alkaloid content of the collected algae, we found that a moderate to high content, ranging from 0.27 ± 0.08 to 3.05 ± 0.31 mg AE/gdw in green algae, 0.75 ± 0.08 to 2.25 ± 0.27 mg AE/gdw in brown algae, and 0.23 ± 0.11 to 2.48 ± 0.08 mg AE/gdw in red algae (Table 2).

Algae vary in their total sterol content and in the variety of sterols present [44]. We found that green algae contained the highest sterol content, followed by that in brown algae and then in red algae (Table 2). *C. prolifera* had the highest value from chlorophytes of 95.35 mg EE/gdw, while *D. dichotoma* and *S. hornschuchii* had approximately 90.10 mg EE/gdw. *A. taxiformis* had 76.45 mg EE/gdw from red seaweeds.

## 4. Conclusion

Seaweeds from the western coast of Libya have similar nutritional values to those found in vegetables and other seaweeds around the world. Hence, we suggest that the studied algal species could be used as alternative nutrient sources for carbohydrate, protein, and lipids for human and animal consumption as these species had a high carbohydrate and low lipid content with an important fraction of protein indicating that marine algae could be considered as healthy food.

## Figures and Tables

**Table 1:** Qualitative phytochemical analysis of crude extracts of green, brown, and red algae.

Seaweed species	Phe	Tan	Phl	Fla	Alk	Sap	Ste	Ter	Cou	Qui	Gly
Green algae, Chlorophyta											
*Caulerpa prolifera*	++	+++	+	+++	++	++	++	+++	+++	−	+
*Codium tomentosum*	++	+	*−*	++	+	+	+	++	+	*−*	+
*Ulva compressa*	+	+	+	+	+	−	++	+	+	−	+
*Ulva intestinalis*	++	++	−	++	++	++	+	++	+	+	+
*Ulva linza*	+	+	−	++	+	+	−	+	+	−	+
*Flabellia petiolata*	+	++	+	+++	+	+	+	+	+++	−	+
*Halimeda tuna*	++	+++	−	+++	+	++	++	+	++	−	+
*Ulva lactuca*	+	+++	−	+	+	+	−	++	+	−	+
Brown algae, Phaeophyceae											
*Cladostephus spongiosus*	++	++	++	+	++	−	++	+++	+	+	+
*Cystoseira compressa*	++	++	++	+++	++	−	++	+++	+++	+	+
*Ericaria amentacea*	+	++	++	++	−	++	++	++	+	−	+
*Dictyota dichotoma*	+++	+++	++	++	+	−	++	+	+++	+	+
*Halopteris scoparia*	++	++	++	+++	+	−	+	+++	+	+	+
*Padina pavonica*	++	+++	+	++	−	+++	+	++	+	−	+
*Petalonia fascia*	++	+++	+++	+++	+	−	++	+++	+++	+	+
*Sargassum hornschuchii*	++	++	++	+++	++	−	++	++	+++	+	+
Red algae, Rhodophyta											
*Asparagopsis taxiformis*	+	++	+	++	+	+	++	+++	++	+	+
*Ceramium virgatum*	+	++	+	+	+	+	+	++	+	−	+
*Corallina officinalis*	+	+	+	+	++	−	−	++	+	−	+
*Pterocladiella capillacea*	+	+	−	++	++	−	−	+	+	−	+
*Gracilariopsis longissima*	+	++	+	+	+	+	+	++	+	+	+
*Hypnea musciformis*	++	++	+	+	++	++	+	++	+	−	+
*Jania rubens*	+	+	+	+	++	−	+	++	+	−	+
*Osmundea pinnatifida*	+++	++	+	+++	++	+	++	++	+	−	+

[+++: high presence; ++: moderate presence; +: low presence; –: absence]. Polyphenols: Phe, tannins: Tan, flavonoids: Flv, alkaloids: Alk, sterols: Ste, saponins: Sap, coumarins: Cou, quinones: Qui, glycosides: Gly.

**Table 2:** Bioactive contents.

Seaweed species	Phenols (mg GAE/gdw)	Flavonoids (mg RE/gdw)	Tannins (mg TAE/gdw)	Alkaloids (mg AE/gdw)	Sterols (mg EE/gdw)
Green algae, Chlorophyta					
*Caulerpa prolifera*	3.46 ± 0.22	24.38 ± 2.07	10.38 ± 1.04	3.05 ± 0.31	95.35 ± 1.44
*Codium tomentosum*	0.70 ± 0.03	6.33 ± 0.14	1.21 ± 0.07	0.89 ± 0.14	30.52 ± 0.93
*Ulva compressa*	0.66 ± 0.03	7.95 ± 0.48	1.51 ± 0.07	0.93 ± 0.11	34.72 ± 4.15
*Ulva intestinalis*	0.70 ± 0.08	6.25 ± 0.33	1.22 ± 0.05	0.54 ± 0.13	50.64 ± 1.07
*Ulva linza*	0.69 ± 0.04	6.77 ± 0.08	1.27 ± 0.06	0.27 ± 0.08	16.39 ± 0.85
*Flabellia petiolata*	0.96 ± 0.03	29.11 ± 0.18	5.60 ± 0.04	1.02 ± 0.16	22.77 ± 2.70
*Halimeda tuna*	2.35 ± 0.15	15.00 ± 0.14	4.02 ± 0.31	1.62 ± 0.17	62.27 ± 4.70
*Ulva lactuca*	0.66 ± 0.03	4.78 ± 0.21	1.18 ± 0.02	0.82 ± 0.08	16.96 ± 2.08
Brown algae, Phaeophyceae					
*Cladostephus spongiosus*	2.01 ± 0.02	7.34 ± 0.46	1.72 ± 0.08	0.79 ± 0.16	34.79 ± 3.09
*Cystoseira compressa*	2.20 ± 0.16	7.41 ± 0.06	1.16 ± 0.07	1.18 ± 0.14	48.67 ± 3.81
*Ericaria amentacea*	0.65 ± 0.05	8.43 ± 0.99	0.87 ± 0.04	1.71 ± 0.27	22.42 ± 1.25
*Dictyota dichotoma*	3.31 ± 0.10	32.38 ± 0.60	4.94 ± 0.46	2.25 ± 0.27	90.10 ± 8.61
*Halopteris scoparia*	1.37 ± 0.06	8.28 ± 0.33	3.47 ± 0.06	0.75 ± 0.08	37.95 ± 2.30
*Padina pavonica*	0.92 ± 0.05	6.86 ± 0.11	2.51 ± 0.13	0.84 ± 0.14	23.65 ± 1.38
*Petalonia fascia*	1.56 ± 0.07	13.49 ± 0.64	3.21 ± 0.10	1.27 ± 0.19	46.92 ± 3.26
*Sargassum hornschuchii*	1.15 ± 0.11	9.00 ± 1.00	1.44 ± 0.14	1.12 ± 0.25	89.97 ± 0.40
Red algae, Rhodophyta					
*Asparagopsis taxiformis*	1.07 ± 0.02	7.40 ± 0.36	0.44 ± 0.04	0.73 ± 0.11	76.45 ± 2.70
*Ceramium virgatum*	0.90 ± 0.03	1.60 ± 0.07	0.05 ± 0.03	1.00 ± 0.04	19.01 ± 1.62
*Corallina officinalis*	0.80 ± 0.02	2.26 ± 0.12	2.01 ± 0.06	2.27 ± 0.45	17.48 ± 0.90
*Pterocladiella capillacea*	0.67 ± 0.03	1.89 ± 0.11	3.24 ± 0.08	0.23 ± 0.11	15.86 ± 0.53
*Gracilariopsis longissima*	0.68 ± 0.03	2.11 ± 0.25	1.54 ± 0.08	1.11 ± 0.05	39.83 ± 2.44
*Hypnea musciformis*	1.05 ± 0.02	2.29 ± 0.28	1.25 ± 0.02	2.48 ± 0.08	41.28 ± 1.71
*Jania rubens*	0.54 ± 0.03	0.49 ± 0.07	0.04 ± 0.02	1.07 ± 0.11	22.12 ± 1.54
*Osmundea pinnatifida*	3.35 ± 0.17	14.84 ± 0.39	3.11 ± 0.09	1.48 ± 0.14	74.04 ± 5.27

## Data Availability

The data that support the findings of this study are available from the corresponding author.
